# S2k guideline occupational skin products: skin protection creams, skin cleansing and skin care products

**DOI:** 10.1111/ddg.70005x

**Published:** 2026-03-16

**Authors:** Michal Gina, Manigé Fartasch, Andrea Bauer, Hans Drexler, Peter Elsner, Johannes Geier, Swen Malte John, Sybille Schliemann, Christiane Altenburg, Richard Brans, Dominique Jasmin Lunter, Alexander Nast, Birgit Pieper, Axel Schlieter, Petra Staubach, Anja Thielitz, Christoph Zeyen

**Affiliations:** ^1^ Institute for Prevention and Occupational Medicine of the German Social Accident Insurance, Institute of the Ruhr University Bochum (IPA) Section Occupational Dermatology Bochum Germany; ^2^ Department of Dermatology University Hospital Carl Gustav Carus Technical University Dresden Dresden Germany; ^3^ Institute and Outpatient Clinic of Occupational Social and Environmental Medicine Friedrich Alexander University Erlangen‐Nürnberg Erlangen Germany; ^4^ Dermatology and Allergology Office SRH Hospital Gera Germany; ^5^ Information Network of Departments of Dermatology (IVDK) University Medical Center Göttingen Göttingen Germany; ^6^ Institute for Interdisciplinary Dermatological Prevention and Rehabilitation (iDerm) Osnabrück University Osnabrück and Department of Dermatology Environmental Medicine and Health Theory Osnabrück University Osnabrück Germany; ^7^ Department of Occupational Medicine Hazardous Substances and Public Health (AGG) Institution for Statutory Accident Insurance and Prevention in the Healthcare and Welfare Services (BGW) Hamburg Germany; ^8^ Department of Pharmaceutical Technology Division Pharmacy Faculty of Science Eberhard Karls University Tübingen Tübingen Germany; ^9^ Division of Evidence‐Based Medicine (dEBM) Department of Dermatology Venereology and Allergology Charité – Universitätsmedizin Berlin corporate member of Freie Universität Berlin and Humboldt‐Universität zu Berlin Berlin Germany; ^10^ Institution for the Woodworking and Metalworking Industries (BGHM) Division Personal Protective Equipment Section Skin Protection German Social Accident Insurance (DGUV) Dortmund Germany; ^11^ Occupational Health BASF Coatings GmbH Münster Germany; ^12^ Department of Dermatology University Medicine Mainz Mainz Germany; ^13^ Dermatology Office Haldensleben Germany

**Keywords:** barrier creams/skin protection creams, Occupational skin products, prevention, skin care products, skin cleansing

## Abstract

The AWMF S1 guideline 013/056 “Occupational Skin Products: Protective Creams, Skin Cleansers and Skin Care Products” from 2014 has been revised and upgraded to S2K level. The aim was to develop a decision aid for the optimal use of occupational skin products for the prevention of hand eczema in the workplace.

The multidisciplinary expert commission included representatives from dermatology, occupational medicine and dermopharmaceutical societies, as well as from German Social Accident Insurance. The update was conducted through a non‐systematic review of current (inter)national scientific literature, focusing on randomized controlled clinical trials.

The revised guideline integrates: *(1)* epidemiological studies demonstrating the effectiveness of skin protection and care, *(2)* results from the Cochrane Review on the primary prevention of occupational hand eczema, *(3)* skin physiological multicenter studies on the application and benefits of skin protection creams and the tolerability of skin cleansing products, *(4)* specific investigations on wet work, and *(5)* current positions on aluminum chlorohydrate and contact allergens based on opinions of the Scientific Committee on Consumer Safety (SCCS) and data from the Information Network of Departments of Dermatology (IVDK).

The recommendations and key statements were agreed upon in a structured consensus conference using a nominal group process under professional moderation. The S2K guideline aims to improve the prevention of occupational hand eczema and promote the health of employees.

## INTRODUCTION

This guideline presents an update and an *upgrade* of the previous S1 guideline on occupational skin products, AWMF registry number 013/056, which was published by the Working Group for Occupational and Environmental Dermatology (ABD) of the German Dermatological Society and the German Society of Occupational and Environmental Medicine (DGAUM) in 2009^1^ and was last updated in 2014.^2^ The guideline will address skin protection, skin care, and skin cleansing products (collectively referred to as occupational skin products) provided by the employer to employees in the framework of an integrated skin protection concept in order to prevent occupation‐related skin diseases.^3^ Occupational skin products belong to the so‐called personal protective measures and are, as cosmetics, in the legal sense subject to the EU regulation on cosmetic products, chapter 5.

This guideline is based on current scientific knowledge according to the national and international literature. The upgrade was conducted taking the previous version and other current European evidence‐ and consensus‐based guidelines into account. In addition, a narrative appraisal of the available inter(national) scientific literature was performed.

For numerous reasons (promotion of compliance, cost‐benefit analysis, potential risks of preventive measures), care should be taken to recommend only preventive measures with scientifically validated efficacy. Given that scientific evidence of effectiveness is currently only available for a fraction of occupational skin products, consensus‐based recommendations based on expert consensus are provided. It is, however, the explicit aim to perform an individual analysis of skin products concerning their efficacy using the methods mentioned in Chapter 3.

In addition to this guideline, we refer to the legal framework provided by the EU regulation on cosmetic products and the German Technical Rules for Hazardous Substances (TRGS) 401 “*Gefährdung durch Hautkontakt, Ermittlung – Beurteilung – Maßnahmen*” (risks resulting from skin contact, identification – assessment – measures) published by the German Federal Ministry of Labor and Social Affairs. Moreover, we refer to the DGUV information 212‐017 “*Auswahl, Bereitstellung und Benutzung von beruflichen Hautmitteln*” (selection, provisioning, and use of occupational skin products) of the German Social Accident Insurance (DGUV).

*For the sections “Notes on using the guideline/Disclaimer” (these apply equally to this short version), “Funding”, “Scope, target user group, and purpose of the guideline”, and “Targeted audience/Users”, see the long version*.

### Nomenclature

The guideline commission has agreed on using the term “skin products” in this guideline. This comprises topical products for skin protection, skin care, and skin cleansing.

### Recommendation strengths, wording, and symbols

Wording, symbols and notes on the interpretation of recommendation strengths are presented in Table 1.

**TABLE 1:** Recommendation strengths – wording, symbolism and interpretation (modified after Kaminski‐Hartenthaler et al., 2014^4^).

Recommendation strength	Wording	Symbol	Interpretation
*Strong* recommendation *for* the use of a procedure	“We recommend…”	↑↑	We believe that all or almost all informed people would make that choice. Clinicians will not have to spend as much time on the process of decision‐making with the patients. In most clinical situations, the recommendation can be adopted as a policy.
Recommendation *for* the use of a procedure	“We suggest…”	↑	We believe that most informed people would make that choice, but a substantial number would not. Clinicians and other health care providers will need to devote more time to ensure that the choice of the procedure together with the potential consequences reflects the values and preferences of individual patients. Decision processes in the health care system require deeper discussions and involvement of many stakeholders.
*Open recommendation* with respect to a procedure	“… may be considered …”	<–>	Currently, no recommendation in favor or against a specific procedure can be made due to certain reasons (for example, no evidence data available, unclear or unfavorable risk‐benefit balance, etc.)
Recommendation *against* the use of a procedure	“We suggest against …”	↓	We believe that most informed people would make that choice, but a substantial number would not.
*Strong* recommendation *against* the use of a procedure	“We recommend against …”	↓↓	We believe that all or almost all informed people would make that choice.

## OCCUPATIONAL SKIN PRODUCTS – DEFINITION AND RELEVANCE IN THE WORKPLACE

In this guideline, the term occupational skin products refers to skin protection, skin care, and skin cleansing products for occupational use.

More specifically, the term “skin protection products” refers to externally applied products intended to protect the skin against irritation in the workplace (barrier creams).^5^, ^6^ Within the concept of integrative skin protection (also called 3‐pillar model), suitable products should be applied in the workplace before starting work and during work; skin protection creams (PCs) are supplemented by so‐called mild skin cleansing products intended to gently remove dirt and aggressive substances from the skin and by skin care products, that is, products that may contribute to regeneration of the epidermal skin barrier (care/regeneration).^7^, ^8^, ^9^ However, from the manufacturer's viewpoint, the term “skin protection product” refers not only to products used to reduce potential irritations in the workplace, but also to products facilitating, for example, cleansing of the skin by their prior application. Given that there are currently no validated scientific studies and classifications concerning these products, they are not addressed in this guideline.

Some additives typically used in skin care products are not suitable for use in PC; this applies, for example, to urea, which is not recommended before and during work activities because of its potential penetration‐promoting effect.

The primary scope of the application of occupational skin products is the prevention of irritant contact dermatitis (synonym: subtoxic cumulative contact dermatitis) that develops frequently at workplaces with repetitive exposure to weak irritants and due to wet work.

In occupations involving a skin hazard, hand eczemas occur with a point prevalence of up to 40%.^10^ In high‐risk occupations, skin hazards should be evaluated and addressed during the health impact assessment. Depending on the intensity of wet work (in the sense of TRGS 401), for example, occupational‐medical health examinations (Ordinance on Preventive Occupational Health Care [*Verordnung zur arbeitsmedizinischen Vorsorge*, ArbMedVV]) should be performed including an explanation of preventive measures. These examinations should include appropriate advice on skin‐friendly work methods and use of skin products in the workplace as well as an explanation of the skin protection plan specifying the use of skin products in the workplace (see Chapter 4).^3^

## EFFICACY OF SKIN PROTECTION CREAMS, SKIN CARE PRODUCTS, AND SKIN CLEANSERS

A recent Cochrane review with meta‐analysis of randomized controlled trials on primary prevention of irritant hand eczema demonstrated that regular application of skin care products, either alone (relative risk [RR] 0.71; 95% confidence interval [CI] 0.46–1.09) or in combination with PCs (RR 0.68; 95% CI 0.33–1.42), showed a tendency toward risk reduction, although this effect did not reach statistical significance. Similar results were obtained for the analysis of health education measures, which were also not statistically significant (RR 0.76, 95% CI 0.54–1.08). Overall, the published evidence on PCs and skin care products or health education measures is insufficient to reliably assess the efficacy of measures for primary prevention.^11^

### Evidence of efficacy of skin protection creams

The efficacy of a PC cannot be assessed based on theoretical consideration of the respective galenic system, its hydrophilic or lipophilic characteristics, or the pH. Given that organizational and methodical challenges and the required resources preclude the conduction of randomized controlled trials/interventional studies under workplace conditions for each substance and each specific exposure,^5^, ^12^ model experiments under idealized conditions are performed to establish efficacy. In these experiments, the efficacy of a PC is quantified by evaluating the reduction of an irritative effect on the skin in vivo by visual assessment and, very often, by bioengineering techniques. However, the informative value of results obtained with model‐based irritation tests is limited, given that working environments are often characterized by complex exposure to a multitude of both hydrophilic and lipophilic occupational substances, which may damage the skin either on their own and/or in combination.^5^, ^13^, ^14^

In context of the joint research project FP 275, a standardized test procedure was developed for tests of hydrophilic irritants (surfactant, acidic, and alkaline range).^15^, ^16^ Based on these findings, “the principles for testing and certification of efficacy of PCs” of the German Social Accident Insurance (DGUV) were formulated.^17^, ^18^ The method verifies the efficacy of PCs against sodium lauryl sulphate – an anionic detergent – as model substance of water‐soluble skin irritants. Once the efficacy of a PC has been established, it receives the DGUV certification mark GS‐PS‐14 “efficacy verified” that can be affixed to the product. PCs labeled with this certification mark have demonstrated their efficacy against the model irritant and provide protection against aqueous substances, such as soaps (typical wet workplaces such as those in healthcare). As yet, a protective effect against lipophilic irritants has not been demonstrated for any substance.^19^

In case of an existing sensitization, the benefit of skin products for prevention of allergic contact dermatitis must be viewed critically.^20^

### Evidence of efficacy of skin care products

According to their manufacturers, skin care products should contribute to the “promotion of regeneration” of the skin after work. Concerning the selection of skin care products, the guideline group has no knowledge of uniform standardized test protocols demonstrating a regenerative effect of skin care products on barrier function. Studies found in the literature investigated the efficacy of care products on various acute and cumulative irritations under conditions of daily exposure.^21^, ^22^, ^23^, ^24^, ^25^, ^26^, ^27^ In addition to a regenerative effect on barrier function, a protective effect was found for some skin care products.^28^, ^29^ While experimental tests demonstrating a regenerative effect of skin care products have been developed,^30^ these have not yet been validated.

Apart from smoothing the skin surface and providing a subjective sense of care, skin care products may also increase the lipid and water content of the epidermis. In addition, they reduce the dryness of the skin after repeated hand washing.^31^ Skin care products should be used in case of beginning erythema/scaling (signs of incipient hand eczema), given that they constitute the required basic therapy of hand eczema according to the guideline on “diagnosis, prevention, and therapy of hand eczema” (registry number 013‐053).^32^, ^33^

### Evidence of efficacy of skin cleansers

Adequate occupational skin cleansing is important for prevention of occupational skin diseases. Skin cleansing is adequate if it removes contaminations, irritants, and allergens effectively from the skin surface while being gentle to the skin. Comprehensible methods and results of the product tests should be available for the user.

Transferable test procedures for the assessment of efficacy and safety of occupational skin cleansers have been developed in the joint research project FP 276 by standardization of model dirts,^34^ development of model hand cleansers, and designing a cleansing device.^35^, ^36^ This method for determining the cleansing effect was evaluated in several centers.^37^, ^38^, ^39^ In future, these test procedures will be suitable for standardized testing of occupational skin cleansers in comparison with the developed model hand cleansers with the aim of contributing to better product transparency.

### Recommendations (evidence of efficacy)

**Table:** 

During selection of skin protection creams for protection against water‐soluble substances, we *suggest* using preferentially skin protection creams whose clinical efficacy has been verified experimentally by standardized test procedures (see, for example, protocols of the multicenter trial FP275, DGUV certification mark).	**↑**	100% (12/12) strong consensus
During selection of occupational skin cleansing products, we *suggest* using preferentially those products, if possible, whose efficacy and tolerability have been demonstrated in vivo by cumulative test procedures (for example, test procedures from the project FP276).	**↑**	100% (11/11) strong consensus *1 abstention due to COI

## PROVISIONING OF SKIN PROTECTION CREAMS, SKIN CARE PRODUCTS, AND SKIN CLEANSERS IN THE WORKPLACE

### Selection of skin protection creams and skin care products

The selection of skin products should be based on a risk assessment taking workplace‐related exposures and risks into account (see Chapter 5). The classical fields of application for PCs are wet work and activities with weak irritants, if use of protective gloves is not possible or not allowed.^3^ PCs offer insufficient protection against contaminations with an often complex composition that may contain hazardous substances absorbed through the skin. In this case, use of protective gloves is mandatory.

The current condition of the skin is of particular relevance when selecting skin care products. Generally, skin care creams are preferred in practice. While formulations with higher lipid content may be used for very dry skin, ointments as anhydrous bases are inappropriate and should be used only shortly, if at all.^40^ According to the AWMF S2k guideline registry number 013‐092 (“guidelines for the use of topical preparations on the skin”), those preparations that take the water content (hydration) of the skin into account and contain hydrophilic substances as additives (urea and/or glycerol) are recommended as basic therapy for improving the barrier function.^40^ If the damage of the barrier is more severe, initiation of additional measures according to the S2k guideline “diagnosis, prevention, and therapy of hand eczema” is recommended.^32^

**Skin protection creams and liquid‐tight gloves**:

Liquid‐tight protective gloves (LPGs) according to TRGS 401 are specialized gloves providing comprehensive protection against liquids. They include chemical‐resistant protective gloves according to DIN EN ISO 374‐1, medical gloves according to DIN EN 455, and fully coated fabric gloves, made, for example, of nitrile rubber. Especially in workplaces with use of LPGs, regular hand washing, and additional contact to water or irritants, a negative impact on the skin barrier must be assumed. If PCs are used together with LPGs, they should not be used at the same time as the protective gloves as they may influence the properties of the gloves.^3^, ^41^ Therefore, if LPGs are used after applying the cream, complete absorption of the PC is mandatory. Currently, there is no conclusive evidence regarding the question whether application of PCs under gloves has a positive impact on the glove‐related occlusion effect. A recent experimental study indicates that application of PCs before the use of LPGs could not prevent the assumed occlusion effect and that some PCs may have even increased the sensitivity of the skin to surfactants.^42^

If wearing of protective gloves is not allowed, for example, during work with rotating parts, the TRGS 401 allows the use of PCs for protection against weak irritants. However, it is important to note that PCs cannot offer the same protection as LPGs.

According to the information of the manufacturers, some PCs may facilitate cleansing and may, therefore, reduce intensity and frequency of aggressive skin cleansing procedures and cleansing intensity, in theory. As yet, no scientific studies quantifying the facilitation of cleansing by these PCs are known to the guideline group. Because of their high content of emulsifiers, these products should not be used under gloves.^3^, ^43^

### Recommendations (selection of skin protection creams and skin care products)

**Table:** 

We *recommend* basing the selection and type of application of skin products on a workplace‐ and activity‐related risk assessment.	**↑↑**	100% (12/12) strong consensus
We *suggest* using skin protection creams with proven protective effect against the aqueous irritants present in the workplace.	**↑**	100% (12/12) strong consensus
We *recommend against* replacing protective gloves by skin protection creams.	**↓↓**	100% (12/12) strong consensus
We *suggest* taking the current condition of the skin, skin dryness, and individual predispositions or previous diseases into account when selecting the skin products.	**↑**	100% (12/12) strong consensus

### Use of skin protection creams and skin care products

PCs and skin care products should be applied on dry and clean skin.^3^ The application times of PCs and skin care products should be considered in the respective workflows. The question of sufficient and optimal frequency of application cannot be answered in general terms and has not yet been sufficiently addressed in scientific studies. It should rather be guided by risk assessment, formulation of the PC, and individual factors. According to the recommendations of the manufacturers, PCs should be applied before each exposure to noxious skin irritants and after each work break or after a specific period (for example, after half a work shift), provided there are no work‐related reasons against this use. In contrast, skin care products can be applied during longer breaks or after work.^44^

So far, the impact of applying a sufficient quantity ofPCs on the quality of the protective effect has only been studies under experimental conditions.^39^ Studies under work conditions on nursing staff have shown that the chosen quantity of PCs is often very small and may not allow for an optimal protective effect.^45^

In practice, the so‐called fingertip unit (FTU) is used to estimate the sufficient quantity of the PC. This is a unit used to determine the quantity of ointments or creams, measured from the fingertip to the distal skin crease of the finger. According to general consensus, one FTU per hand can be recommended for the majority of PCs and care creams (formulation: cream).

While the use of PCms for facilitating skin cleansing results in removal of contaminations that is more gentle to the skin, they will not necessarily protect against the components of the dirt (on this aspect, see also explanations above). If possible, protective gloves should be used for activities associated with contamination.

For hygienic and galenic reasons, the expiration date of a skin product (for example, in dispenser systems) and its consistency (for example, phase separation) must be observed in occupational medical practice.

To avoid microbiological contamination, single‐use dispensers or press‐type dispenser systems are recommended. According to the S2k guideline on hand antisepsis and hand hygiene (registry number 075‐004), PCs and skin care products should be provided in dispensers or tubes due to the risk of contamination.^46^ For hygienic reasons, the use of pots is not recommended.

In association with a recognized occupational disease (*Berufskrankheit*, BK) 5101 or already in case of an impending risk of developing a BK (in context of measures related to § 3 or dermatologist's report), skin care products may also be prescribed by physicians as basic therapeutics for individuals at the expense of the respective accident insurance institution, if a corresponding application for treatment exists. It must be noted that medically prescribed skin products should be used in the workplace in coordination with the employer. Moreover, coordination with the occupational safety officer or – if possible – the company occupational physician is recommended.

### Recommendations (use of skin protection creams and skin care products)

**Table:** 

We *recommend* applying skin protection creams and skin care products in sufficient quantity and frequency. In this context, we *recommend* to take the product‐specific recommendations into account.	**↑↑**	100% (13/13) strong consensus
For the majority of skin protection creams and skin care creams, we *suggest* using the quantity of at least one fingertip unit (FTU) for the surface of one hand.	**↑**	100% (12/12) strong consensus
We *suggest* applying a skin protection cream before the skin‐damaging activity – if indicated according to risk assessment.	**↑**	100% (13/13) strong consensus
We *recommend* applying skin care products after work.	**↑↑**	100% (13/13) strong consensus
We *suggest against* using skin protection creams immediately before donning liquid‐tight protective gloves, if possible, given that they may affect, among other factors, the properties of the gloves.	**↓**	100% (13/13) strong consensus

### Selection and use of skin cleansers

Selection and composition of skin cleansers depend, in general, on the type of contamination (simple, gross, special) while a corresponding step‐by‐step plan depending on the degree of contamination must be observed. The intensity of cleansing and the selection of the cleanser must be adjusted to the degree of contamination.^3^ When using skin cleansers, it must be taken into account that products with comparable cleansing effect may have major differences with respect to skin tolerability.^47^

Hand cleansing has been shown to result in damage of the skin barrier and, if the frequency of hand washing is too high, in irritant contact dermatitis.^48^, ^49^, ^50^ From a preventive view, frequency of washing,^51^ duration of washing, composition of cleansers,^52^ and use of auxiliary tools (for example, hand brushes) should be taken into consideration.^53^ The frequency of hand washing should be reduced to a minimum, in so far as this complies with hygiene regulations. Hand washing is especially relevant in case of visible contaminations. According to current knowledge, the irritative potency, from a chemical perspective, is primarily dependent on the used type of detergent (anionic, cationic, amphoteric, non‐ionic).^54^, ^55^, ^56^ It is not known for certain which role the pH plays in skin tolerability of skin cleansers. Previous studies have shown that cleansers should have a neutral or acidic pH rather than an alkaline pH.^54^, ^57^, ^58^, ^59^, ^60^, ^61^

In practice, mild cleansing products without abrasive agents should be preferred.^3^

If heavy contaminations cannot be eliminated by mild cleansers, products containing abrasive agents may be used. Cleansing of hands with products containing abrasive agents should be reduced where possible to a minimum, for example, at the end of the workday. The use of brushes should be avoided, if possible. Heavy contamination may already be reduced with paper towels, thus avoiding the use of brushes. For this purpose, modern abrasive agents made from biologic materials should be preferred.^62^, ^63^ The use of hard and abrasive brushes is not recommended.^53^

In general, the use of antiseptic soaps is not recommended. They cannot replace hand disinfection by alcohol‐based hand disinfectants.^64^

### Recommendations (selection and use of skin cleansers)

**Table:** 

When selecting the skin cleanser, we *recommend* its adjustment to the degree of contamination and the condition of the skin.	**↑↑**	91.7% (12/13) consensus
We *recommend* reducing the frequency of hand cleansing to the necessary minimum.	**↑↑**	100% (13/13) strong consensus

## SAFETY ASSESSMENT AND ADVERSE EFFECTS

### Safety assessment in cosmetology

In Germany, occupational skin products in the sense of this guideline are legally regulated as cosmetics. According to Regulation (EC) No 1223/2009 of the European Parliament and the Council from November 30, 2009 (“EU regulation on cosmetic products”), cosmetic products are defined as “any substance or mixture intended to be placed in contact with the external parts of the human body (epidermis, hair system, nails, lips and external genital organs) or with the teeth and the mucous membranes of the oral cavity with a view exclusively or mainly to cleaning them, perfuming them, changing their appearance, protecting them, keeping them in good condition or correcting body odours”.^65^ The regulation is directly applicable in all member states of the European Union and, therefore, also in Germany.

According to the EU regulation on cosmetic products, “cosmetic products must be safe for human health when used under normal or reasonably foreseeable conditions of use”. Requirements for the safety assessment of cosmetic ingredients have been presented by the *Scientific Committee on Consumer Safety* (SCCS), which is active on behalf of the EU commission; these are updated on a regular basis.^66^

### Sensitizations caused by skin products

As cosmetic products, PCs are subject to the regulation on cosmetic products (EC No 1223/2009 2023), which is permanently adjusted to the latest scientific knowledge in accordance with respective EU regulations. Recently, for example, the list of declarable fragrances was expanded to 81 substances requiring labeling by EU Regulation 2023/1545.

Given that skin products are also applied to pre‐damaged skin, the risk of sensitization to individual ingredients is enhanced, in theory. In the scientific literature, however, only individual case reports, but no recent corresponding studies on larger patient groups are available. Studies on sensitizations caused by skin products are impeded by the fact that ingredients of skin products used at work are not fundamentally different from cosmetic products used privately. Accordingly, it is usually impossible to clarify how an identified sensitization was acquired.

In the guideline group, arguments in favor and against the use of fragrance‐containing skin products were discussed with respect to sensitization and irritation. Based on the currently available studies and the experience from daily clinical practice, the guideline group is not able to formulate any recommendation on this complex topic, because no consensus was reached. Further research on the use of fragrances in skin products is required in order to formulate a recommendation.

Table 2 shows substances commonly associated with contact allergies in studies that may be used in cosmetic products. The focus is on preservatives, fragrances, and natural extracts. Primary criterion was the reaction rate of more than 1% (including targeted patch tests). Table 2 makes no claim to completeness. If the terms are not identical, the names according to the *International Nomenclature of Cosmetic Ingredients* (INCI) are provided in parentheses.

**TABLE 2:** Ingredients not recommended in occupational skin products due to the increased risk of contact sensitization, particularly in pre‐damaged skin.

Name (INCI *)	
***Preservatives***	100% (12/12) strong consensus 1 abstention due to conflicts of interest
Methylchloroisothiazolinone	
Methylisothiazolinone	
***Fragrances***	
Citral	
Eugenol	
Farnesol	
Geraniol	
Hydroxycitronellal	
Isoeugenol	
Cinnamic aldehyde	
Cinnamic alcohol	
***Extracts of natural products / essential oils***	
Jasmine absolute (Jasminum officinale extract)	
Lemongrass (Cymbopogon schoenanthus oil)	
Clove oil (Eugenia caryophyllus leaf oil)	
Peru balsam/Myroxolon pereira (Balsam Peru)	
Propolis (Propolis extract)	
Sandelwood oil (Santalum album oil)	
Ylang Ylang oil (Cananga odorata flower oil)	

### Recommendations (sensitizations and irritations caused by skin products)

**Table:** 

We *suggest against* using occupational skin products containing the ingredients of Table 2, especially on pre‐damaged skin.	**↓**	100% (11/11) strong consensus
When selecting the skin product, we *recommend* taking individual sensitizations into account.	**↑↑**	100% (12/12) strong consensus

### Potential irritative effects of occupational skin products

*For Chapter 5.4 “Potential irritative effects of occupational skin products”, see long version*.

### Skin cleansing versus skin disinfection in activities posing a risk of infection

According to the information of the manufacturers, hand disinfectants should be distributed over the hands in sufficient quantity (usually 3–5 mL) and left sufficiently long to take effect. Superfluous hand disinfectant should be allowed to evaporate and not be wiped off.^46^

Measures of hand hygiene are aimed at reducing the contamination of the skin with biologic pathogens (viruses, bacteria, or parasites) by their mechanical or chemical detachment; given the presence of the physiologic microbiome, complete sterility of the skin is not feasible.^67^

In recent years, various dermatological research groups^68^, ^69^, ^70^, ^71^ have conducted studies on the irritative potency or skin tolerability of measures of hand hygiene. Among other findings, these studies revealed that detergent‐related effects are enhanced by subsequent glove occlusion, while this was not found for alcohol‐based disinfectants.^71^ The cumulative irritative potency of alcohol‐based disinfectants is low and significantly lower than that of detergents.^2^, ^72^, ^73^ From the view of maintaining dermatological health, hand disinfection is preferred over hand washing in case of non‐visible contamination.^3^, ^68^, ^74^ There is no evidence for reducing alcoholic antisepsis by skin care.^75^

### Recommendations (skin cleansing versus skin disinfection in activities posing a risk of infection)

**Table:** 

In activities posing a risk of infection, we *suggest* preferring disinfection with hand disinfectants over skin cleansing with detergents in case of non‐visible decontamination, if possible.	**↑**	100% (11/11) strong consensus

### Safety assessment of aluminum chlorohydrate in occupational skin products

The German Federal Institute for Risk Assessment (BfR) has revised its safety assessment of aluminum chlorohydrate from 2020.^76^ Taking a new study on the absorption of aluminum into consideration, the absorption of aluminum from antiperspirants via the skin was considerably lower than previously assumed. The contribution of this source of absorption to the entire absorption of aluminum is, therefore, very low.

The Scientific Committee on Consumer Safety (SCCS) of the European Commission has published its assessment of comprehensive new data on the absorption of aluminum chlorohydrate from antiperspirants via the skin. Taking the current human data on dermal absorption of aluminum under realistic conditions of use into account,^77^ the SCCS concludes that users absorb significantly lower amounts of aluminum than assumed on basis of the originally available data.^66^

### Recommendations (safety assessment of aluminum chlorohydrate in occupational skin products)

**Table:** 

The application of a skin protection cream or skin care product with aluminum chlorohydrate *may be considered* if the skin is intact.	**<–>**	81.8% (9/11) Consensus

### Impact of skin protection creams on the penetration of foreign substances

The intact stratum corneum forms the actual barrier against dermal penetration of chemical substances. Only if the stratum corneum has been penetrated can a substance be absorbed.^78^ Apart from that, small molecules, in particular, may enter the skin via the hair follicles.^79^

Penetration of a substance through the skin may be facilitated by the presence of other substances. Additives like emulsifiers are thought to be responsible for the facilitating role of PCs with respect to the penetration of hazardous substances.^80^, ^81^, ^82^, ^83^

PCs should only be used for protecting the skin against irritation and not for protection against absorption of occupational substances with systemic toxicity.

Experimental studies have shown that penetration of hydrophilic and lipophilic solvents is not reduced by pretreating the human skin with PCs,^84^, ^85^ but is usually promoted.^81^, ^82^, ^83^, ^84^, ^86^, ^87^

In summary, it cannot be assumed that application of PCs prior to exposure to foreign substances results in a general reduction of percutaneous absorption of these substances.

### Recommendations (impact of skin protection creams on the penetration of foreign substances)

**Table:** 

We *recommend* considering the risk of a potentially accelerated penetration of hazardous substances/foreign substances by skin protection creams during their use and selection.	**↑↑**	100% (12/12) strong consensus
We *recommend against* using skin protection creams for protection against percutaneous absorption of occupational substances with systemic toxicity, for example, carcinogenic substances or dermally absorbable substances.	**↓↓**	100% (11/11) strong consensus

### Direct contact to patient‐specific care products and critical use of protective gloves in healthcare. Critical use of disposable gloves in healthcare

The German Commission for Hospital Hygiene and Infectious Disease Prevention (KRINKO) emphasizes in a published KRINKO recommendation the necessity of a balanced use of disposable gloves in order to ensure hygienic protection while taking ecological and health‐related aspects into account.^88^ In the authors’ view, however, the KRINKO recommendation includes two activities that should be performed while being protected by liquid‐tight gloves, namely *(1)* body washing and *(2)* applying creams to patients.

**Table:** 

In healthcare, we *suggest* using liquid‐tight gloves during body washing to reduce the skin contact with cleansing products and water.	**↑**	100% (11/11) strong consensus
We *suggest* using liquid‐tight gloves when applying care products to the skin of patients to reduce the direct contact with potentially sensitizing ingredients and the frequency of hand washing.	**↑**	100% (11/11) strong consensus

*For the sections “Documentation recommendations for manufacturers”, “Outlook and research needs”, “Limitations of the guideline”, “Information about this guideline” (including “Handling of conflicts of interest”), and “Methods” (including “Generation of recommendations/Consensus conference”, “Approval of the guideline”, and “Exploitation rights”), see long version*.

## CONFLICT OF INTEREST STATEMENT

A full list of declared conflicts of interests is available in the guideline document at https://register.awmf.org/de/leitlinien/detail/013‐056.
